# The iCRF Generator: Generating interoperable electronic case report forms using online codebooks

**DOI:** 10.12688/f1000research.21576.2

**Published:** 2020-03-23

**Authors:** Sander de Ridder, Jeroen A.M. Beliën

**Affiliations:** 1 Department of Pathology, Amsterdam UMC, Vrije Universiteit Amsterdam, Amsterdam, 1081 HV, The Netherlands

**Keywords:** Interoperability, eCRF, iCRF, Codebook, FAIR, Software, EDC, Clinical data

## Abstract

Semantic interoperability of clinical data is essential to preserve its meaning and intent when the data is exchanged, re-used or integrated with other data. Achieving semantic operability requires the use of a communication standard, such as HL7, as well as (functional) information standards. Manually mapping clinical data to a medical thesaurus, such as SNOMED CT, is complicated and requires expert knowledge of both the dataset, including its context, and the thesaurus. As an alternative, the (re-)use of codebooks, data definitions which may already have been mapped to a thesaurus, can be a viable approach.

We’ve developed the iCRF Generator, a Java program that can generate the core of an interoperable electronic case report form (iCRF) for several of the major electronic data capture systems (EDCs). To build their CRFs, users can select one or more items from established codebooks, available from an online system called ART-DECOR. By providing an easy to use method to create CRFs for multiple EDCs based on the same codebooks, interoperability can be more easily attained.

## Introduction

Clinical data is essential for health research. Traditionally, such data was captured using paper case report forms (CRFs) and entered into a database manually. Nowadays, the data is often captured directly with electronic CRFs (eCRFs) in an electronic data capture (EDC) system. This has improved the quality of the captured data as well as decreased costs for data collection (e.g. 1, 2).

To allow the captured data to be used beyond its original purpose requires the data to be FAIR (Findable, Accessible, Interoperable and Reusable) ^3^. By making data semantically interoperable, it can be exchanged between systems whilst preserving the meaning of the data ^4^. Furthermore, it allows multiple data sources to be combined and understood by computers, thereby e.g. facilitating clinical decision support systems ^5^. Hence, when setting up a new data collection protocol, the eCRF should be designed with interoperability in mind. Achieving semantic interoperability requires the use of a communication standard, such as HL7, as well as (functional) information standards ^4^, such as the NCI thesaurus or SNOMED CT. However, mapping study-specific terminology to a thesaurus requires expert knowledge of the thesaurus, the data and its context. Therefore, reusing existing codebooks from studies and well-known datasets or CRF templates, such as available from CDISC’s CDASH, the Portal of Medical Data Models website and the University of Wisconsin-Madison, can be a viable alternative. Reusing these elements at the very minimum facilitates interoperability with other datasets using these definitions. Furthermore, in many cases well-known codebooks have already been mapped to a thesaurus. For example, in the Basic Health Data Set, which is the standard that will be used by hospitals to exchange healthcare data in the Netherlands (available here, Dutch only), many of the items have been mapped to SNOMED CT.

In this paper, we introduce the iCRF Generator, a program that allows users to easily generate interoperable electronic case report forms (iCRFs) based on online codebooks, thereby improving the interoperability of clinical data collected in and between EDCs. Whereas normally CRF generation is an integrated part of the EDC (e.g. Castor EDC, REDCap), our program can generate the core of a CRF for multiple EDCs. At this time, three systems are supported: Castor, OpenClinica 3 and REDCap. The program allows a user to select one or more codebooks available from an online system called ART-DECOR which allows, amongst others, the storage of dataset definitions, and select items of interest, including their codelists. Hence, if a codebook is mapped to a medical thesaurus, the iCRF Generator allows the user to use these mappings, preventing the labour-intensive manual mapping. The program currently supports six codebooks, which are further described in the Methods section.

## Methods

### Implementation

The iCRF Generator was written in Java 8 and later migrated to Java 12 for JavaFX compatibility. Dependencies are managed using Maven and include: JavaFX and ControlsFX for the UI, Apache POI for Excel file management and Log4j for logging. A ZIP file of the iCRF Generator distribution is available for both Mac and Windows. It includes a Java Runtime Environment to ensure independence of the locally installed Java version and ensures the program works out of the box. Source and distribution files are available on GitHub: https://github.com/aderidder/iCRFGenerator/.

### Supported codebooks

The iCRF Generator is designed to use codebooks defined in ART-DECOR. ART-DECOR is an open-source tool suite that supports the creation and maintenance of HL7 templates and allows the storage of dataset definitions. Nictiz, the centre of expertise for eHealth and the Dutch SNOMED-CT release centre, facilitates ART-DECOR to create health information standards that are publicly accessible. The iCRF Generator currently offers access to six of these codebooks, which were chosen because of their national relevance (codebooks 1, 2 and 3) and our involvement (4, 5 and 6). The number of items mentioned below are estimates, as codebooks in ART-DECOR may inherit items from other codebooks multiple times.

- **1. **The Clinical Building Blocks ( Zorginformatiebouwstenen): information models of minimal clinical concepts. They are used as the basis for the Basic Health Data Set. The 2017 set contains 100 building blocks, with about 940 items and 211 codelists.
- **2. **The Basic Health Data Set ( Basisgegevensset Zorg): codebook used for the standardised exchange of patient data between e.g. healthcare providers. Implementation of this set is prioritised in healthcare systems like electronic health records. The Basic Health Data Set is aligned with the European Patient Summary. The codebook (version 2017) contains 3742 items, of which 876 have codelists.
- **3. **The National Institute for Public Health and the Environment’s national screening codebook of bowel cancer and cervical cancer ( RIVM bevolkingsonderzoeken). This codebook (version 2019) contains 258 items, of which 103 have codelists.
- **4.** Cancer Core Europe: a European cancer research alliance which aims at bringing together the expertise and critical mass necessary to make translational research available in the clinic. This codebook (version 2017) contains 104 items, of which 93 have codelists.
- **5.** The PALGA Colon biopsy protocol: PALGA is the nationwide network and registry of histo- and cytopathology in the Netherlands. This codebook (version 33) contains 70 items, of which 45 have codelists.
- **6. **The PALGA Colorectum carcinoma protocol. This codebook (version 59) contains 198 items, of which 121 have codelists.

Some of the codebooks are available in English, as well as Dutch. 

### Operation

A standard PC or Mac should be able to run the program without any issues. The program was tested on a Windows 7 PC, a Windows 10 PC, a virtual machine with OS X El Capitan, a iMac and a MacBook Pro both running OS Catalina 10.15.3. To give an indication of the program’s memory usage: selecting a single codebook, the program uses around 230 megabytes of memory; increasing this number to eight codebooks increased the memory usage to 420 megabytes. An internet connection is required, as the program retrieves metadata as well as codebooks from ART-DECOR via the REST API.

### Use cases

**Figure 1.**
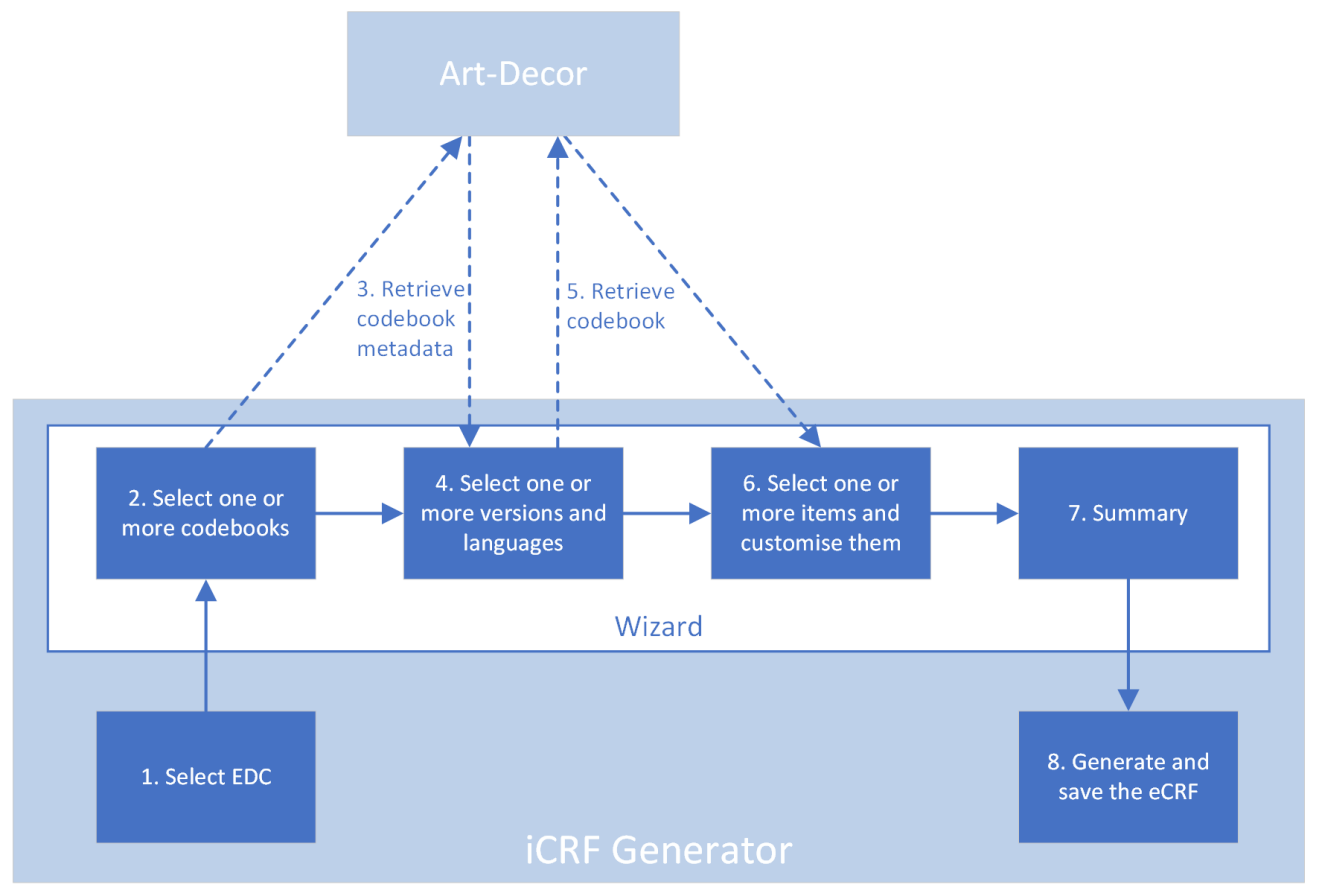
Flowchart showing steps involved in using the iCRF Generator. Dotted lines are calls made by the program to ART-DECOR’s REST services.

**Figure 2.**
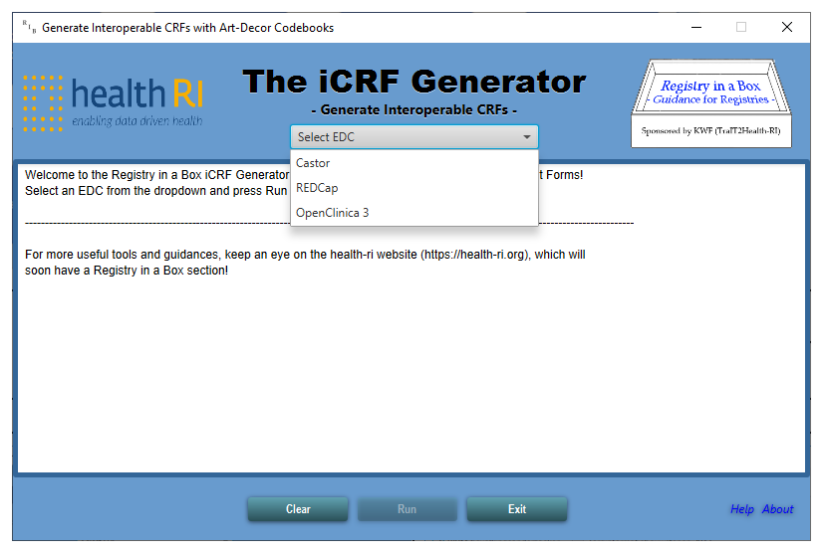
User selects an electronic data capture system (EDC) from the dropdown.

**Figure 3.**
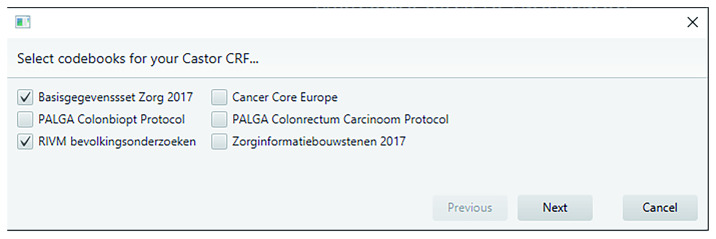
User selects one or more codebooks.

**Figure 4.**
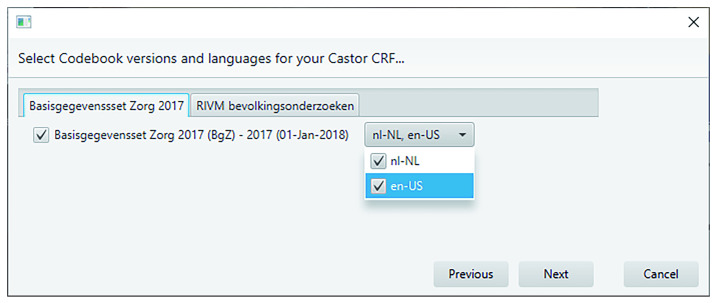
User selects codebook version(s) and language(s).

**Figure 5.**
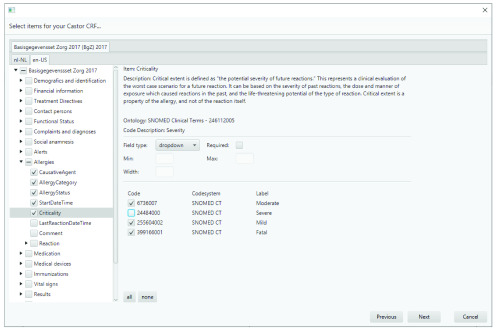
User selects items on the left-hand side in the tree and customises an item’s details in the electronic data capture system (EDC)-specific right-hand side.

**Figure 6.**
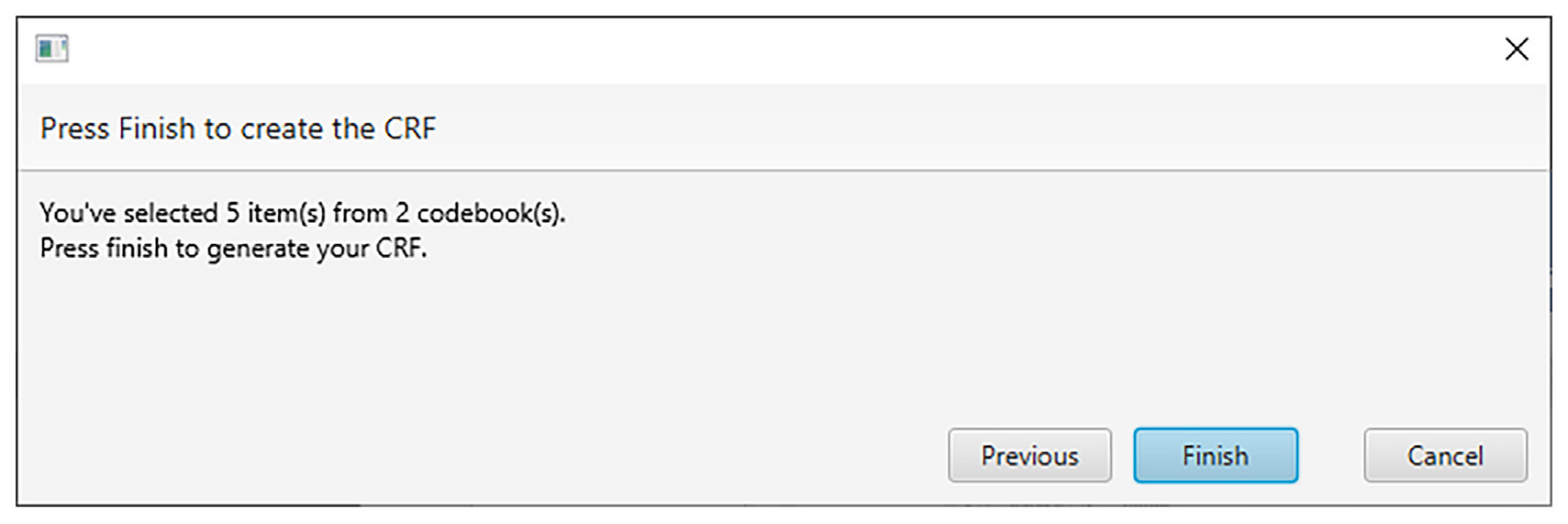
Summary shows number of selected items.

A typical use case for the iCRF Generator is in the design phase of a study or registry. When a decision has been made on what clinical data is going to be collected, the data manager has to design and build the CRFs for data collection. Instead of manually designing the items and mapping them to a medical thesaurus, which takes a lot of time, the iCRF Generator can be used to select items which have already been mapped from the available codebooks and generate the basis of the case report forms. Figure 1 illustrates the iCRF Generator’s complete workflow and Figure 2– Figure 6 show actual examples of the workflow. In a typical use case, the user first selects an EDC from the dropdown ( Figure 1, step 1; Figure 2). The user then clicks the “Run” button, after which the wizard interface is started. The first wizard page asks the user to select one or more codebooks ( Figure 1, step 2; Figure 3). When a user has selected a codebook and presses the “Next” button, a REST-call is made to retrieve an XML which contains which versions and languages are available for the selected codebooks ( Figure 1, step 3). The second page shows this information to the user, allowing selection of the versions and languages of interest ( Figure 1, step 4; Figure 4). When the user proceeds to the next page, the selected codebooks are retrieved via one or more REST-calls ( Figure 1, step 5). These XML files are parsed and the information it contains about the items and their possible values is shown on the third page ( Figure 1, step 6; Figure 5). This EDC-specific page allows users to select and customise the items that have to be included in the CRF. The final page of the wizard shows a short summary of the number of selected items ( Figure 1, step 7; Figure 6). Upon completion of the wizard, the program generates the CRF in the format required by the selected EDC and the file is saved to disk ( Figure 1, step 8). This file can then be imported into the EDC-system or opened in an editor of choice.

## Discussion

To allow for the use of data beyond its original purpose, it is essential that the data is FAIR (Findable, Accessible, Interoperable and Reusable) ^3^. To preserve meaning and intent of clinical data when it is exchanged, requires the data to be semantically interoperable, which requires the use of content standards. Manually mapping data definitions to a medical thesaurus such as SNOMED CT is complicated and time consuming. For many items, however, mappings are already available (e.g. in ART-DECOR, the Portal of Medical Data Models and CDISC’s CDASH). Reusing these definitions (codebooks) can, therefore, be a viable alternative, as interoperability with other datasets using these codebooks is usually easily achieved.

In this paper we introduced the iCRF Generator, a program which can generate electronic case report forms for three major electronic data capture systems: Castor, REDCap and OpenClinica 3. The program allows a user to select items and codelists from several highly relevant codebooks available from the online system ART-DECOR. By providing an easy to use program to generate CRFs using these codebooks, data will be collected using the same definitions, which enhances the interoperability and FAIRness of the data.

### Local caching of codebooks

One important usability aspect of software is the software’s performance. The codebooks available in the iCRF Generator are parsed from XML files generated by ART-DECOR. It takes ART-DECOR around 30 seconds to generate the XML file for the National Institute for Public Health and the Environment screening codebook. Furthermore, in some cases XML files can reference other XML files, which then have to be downloaded and parsed as well. If a user has to wait every time a codebook is selected, user acceptance will quickly erode. Hence, we introduced local caching of downloaded codebooks, which makes usage nearly instantaneous once the codebook is locally available. Furthermore, we intend to make a ZIP file of the cache available for download. Note that downloading a codebook XML from ART-DECOR only takes place if it is accessed for the first time or when a new version of a codebook becomes available and is selected by the user.

### Additional EDCs

The iCRF Generator can easily be expanded to include additional EDCs, such as OpenClinica 4, Research Manager and Alea if there is demand and the import formats are available. Support for CDISC ODM is on our roadmap. If desirable, additional internationally established formats, may also be included in the future.

### Additional codebooks

At this point, the iCRF Generator gives access to six nationally established codebooks, some of which support multiple languages and multiple versions. To improve the user-base for the iCRF Generator, the number and variety of codebooks available must increase. While nationwide standards, such as the Basic Health Dataset, can be readily made available, some form of governance may have to be put in place to establish other types of curated codebooks. This could stimulate the community to help build high-quality codebooks, mapped to medical thesauri, while preventing too much codebook redundancy and conflicting items. Furthermore, internationally established codebooks, such as CDISC’s CDASH can also be made available.

### EDC-specific item customisation within iCRF Generator

When an item is selected in the item tree, a user can customise the item. As each EDC has different requirements for its CRFs, the customisation options we provide vary per EDC. As an example, OpenClinica 3 has a “Field Type” (e.g. “Radio”, “Single-Select”) and a “Data Type” (e.g. “ST”, “INT”), whereas in Castor the data type does not exist as a separate entity.

By adding this EDC-specific customisation, the iCRF Generator’s code is more difficult to maintain. However, by allowing the data manager to customise essential fields, the iCRF Generator can provide a ready-to-use CRF. This enhances the user experience, making it a worthwhile investment. The customisation options we currently provide are limited. The iCRF Generator’s purpose is to facilitate generation of interoperable CRFs. Hence, if everything could be customised, for example replacing the codes in codelists with custom codes, it would undermine the purpose of the program. Furthermore, the iCRF Generator is work-in-progress and some further item customisation may be added in the future.

### Similar work - alternative solutions & templates

A tool somewhat similar to our own is ODMedit ^6^. ODMedit provides a web-based interface to allow users to create a CRF based on elements stored in the Meta Data Repository. When a user has finished creating the CRF, it can either be downloaded in ODM format or uploaded to the Medical Data Models-portal. From there it can be downloaded in multiple formats.

ODMedit differs from our software in several ways. Whereas ODMedit immediately provides access to all items in its repository, we keep our items grouped by codebook. Furthermore, with ODMedit users can immediately add new and edit existing items, and new items are automatically made available in the repository. In our tool we are providing access to only handpicked codebooks, from which the user can select items and customisation of these items is kept to a bare minimum. By allowing users to select items from well-known and supported codebooks only, we believe it should be easier to find the correct item - e.g. if you need pathology definitions, use items from the pathology codebooks. However, we may have to add a search function at some point to make it easier to find items within a codebook. Another difference is that we decided to explicitly ask for which EDC tool the user wishes to create the CRF to allow for EDC specific options. On the other hand, ODMedit does support some features which we do not yet support, such as a repeating group. We may add this at a future time.

An OpenClinica 3 specific CRF generator is also available. This tool converts a csv file to Excel and provides a user with an interface to edit the CRF. However, the tool does not facilitate interoperability.

Multiple initiatives exist that aim at providing templates to improve interoperability. We list several such initiatives below. The National Institute of Health offers Common Data Elements, data elements that are common to multiple data sets across different studies. CDASH, provided by CDISC, gives guidance for developing CRFs used in clinical trials ^7^. The OpenClinica Building Blocks developed by TraIT provide OpenClinica users with templates to which they can add study-specific items and remove items that are not necessary for their study. The Australian Government launched a platform for digital health. They provide an extensive library of documents, tools and much more for implementers and developers. The Global Alliance for Genomics & Health ( GA4GH) has several workstreams, amongst which one for Clinical & Phenotypic Data Capture, that “Supports the clinical adoption of genomics through establishing standard ontologies and information models to describe the clinical phenotype for use in genomic medicine and research, including the capture and exchange of information between electronic clinical systems and research.”

## Software availability

Source code available here: https://github.com/aderidder/iCRFGenerator

Archived source code as at time of publication: https://doi.org/10.5281/zenodo.3563500 ^8^

License: GNU GPL v3 license.
