# Supplementary material for: The iCRF Generator: Generating interoperable electronic case report forms using online codebooks
Source: F1000Res. 2020 Mar 23;9:81. Originally published 2020 Feb 4. [Version 2] doi: 10.12688/f1000research.21576.2 (PMC7291075; doi:10.12688/f1000research.21576.2)
Supplement: Peer review history

# Peer review: Reviewer response for version 2

**DOI:** 10.5256/f1000research.25280.r63637

**Published:** 2020-06-11

**Authors:** Hugo Leroux

**Affiliations:** 1 Australian e-Health Research Centre, Brisbane, Qld, Australia

This paper describes a iCRF generator that is able to generate codebooks that can be used in three EDCs, namely CASTOR, OpenClinica and REDCap.

 This is a very important and useful tool for the clinical research community in promoting and achieving semantic interoperability and the authors should be commended on their effort.

 While the tool offers the ability to map the fields and values to the NCI thesaurus and SNOMED CT terminology, what is not clear to me is whether the user is able to select a particular terminology server to use. This is particularly important for local variants of the SNOMED CT terminology that may contain terms not present in the global SNOMED CT terminology.

 Furthermore, by providing the functionality to connect to a particular terminology server opens up the possibility for terms from other standardised terminologies and ontologies to be included within the codebooks.

 The authors state that “internationally established codebooks, such as CDISC’s CDASH can also be made available”. It would be useful to explicitly state how they propose for this to happen. Will it be posted on a website? Will it be part of the codebook libraries, as is the case with REDCap?

 What is also not clear is whether a codebook created for, say, Castor, can be used for another EDC, say OpenClinica. If so, it would be useful to state how the iCRF generator deals with the different implementations (For a start, REDCap implements a slight variation of CDISC ODM, which is not compatible, out-of-the-box, with OpenClinica, which offers a more consistent implementation of CDISC ODM).

Are the conclusions about the tool and its performance adequately supported by the findings presented in the article?

Yes

Is the rationale for developing the new software tool clearly explained?

Yes

Is the description of the software tool technically sound?

Yes

Are sufficient details of the code, methods and analysis (if applicable) provided to allow replication of the software development and its use by others?

Yes

Is sufficient information provided to allow interpretation of the expected output datasets and any results generated using the tool?

Yes

Reviewer Expertise:

Data science, semantic interoperability and clinical research framework design

I confirm that I have read this submission and believe that I have an appropriate level of expertise to confirm that it is of an acceptable scientific standard.

# Peer review: Reviewer response for version 2

**DOI:** 10.5256/f1000research.25280.r61614

**Published:** 2020-03-26

**Authors:** Rianne Fijten, Petros Kalendralis

**Affiliations:** 1 Department of Radiation Oncology (MAASTRO), GROW School for Oncology and Developmental Biology, Maastricht University Medical Centre (MUMC+), Maastricht, The Netherlands; 2 Department of Radiation Oncology (MAASTRO), GROW School for Oncology and Developmental Biology, Maastricht University Medical Centre (MUMC+), Maastricht, The Netherlands

The authors have thoroughly and sufficiently answered any question or concerns we had.

 They have added a lot more structure to the article, for example adding a clear use-case scenario in which they describe the steps a user should take (including extra figures for each step).

 We are happy to accept this version of the article.

Are the conclusions about the tool and its performance adequately supported by the findings presented in the article?

Partly

Is the rationale for developing the new software tool clearly explained?

Yes

Is the description of the software tool technically sound?

Partly

Are sufficient details of the code, methods and analysis (if applicable) provided to allow replication of the software development and its use by others?

Yes

Is sufficient information provided to allow interpretation of the expected output datasets and any results generated using the tool?

No

Reviewer Expertise:

Our area of research focuses on making medical data FAIR. For example, we employ the Personal Health Train to share information across medical centers in a privacy-preserving manner.

We confirm that we have read this submission and believe that we have an appropriate level of expertise to confirm that it is of an acceptable scientific standard.

# Peer review: Reviewer response for version 1

**DOI:** 10.5256/f1000research.23777.r59545

**Published:** 2020-02-14

**Authors:** Martin Dugas

**Affiliations:** 1 Institute of Medical Informatics, University of Münster, Münster, Germany

The iCRF Generator: Generating interoperable electronic case report forms using online codebooks

 This manuscript is about iCRF generator, a software tool to generate interoperable CRFs.

 To foster re-use of data and implement FAIR principles are important topics.

 Source code of iCRF-generator is available, which is a plus.

 Major comments

 page 2, Methods, Supported Codebooks: Quantitative Information on 6 codebooks is missing: how many item definitions per codebook are available? how many codelist(items)?

 page 2, Introduction: iCRF generates CRFs in Castor, OpenClinica3 and REDCap-Format.

 Why not CDISC ODM, which is endorsed by FDA? The Portal of Medical Data Models provides CRFs in 18 formats

 page 3, Discussion "Manually mapping data definitions to a medical thesaurus such as SNOMED CT is complicated and time consuming"

 => Yes, this is absolutely correct. But it should be mentioned, that this mapping was already done for large sets of data items.

 For example, the portal of medical data models provides ~520.000 data items with manual (physician-based) terminology mappings. The portal of medical data models is not just a website, it is a registered European information infrastructure.

 page 6, Additional EDCs: CDISC ODM is not just an internationally established format, it is required by regulatory authorities (FDA).

 Define XML (FDA) is based on CDISC ODM !

 page 6, EDC-specific item customisation: processing of individual data items is a very work-intensive process, given the high number of data items in clinical studies (~ 500 - 2000). Re-Use of itemgroups (sets of data items) would be useful (this feature is available in ODMedit, which is covered in the discussion)

 page 6, Discussion, similar work: It should be mentioned, that ODMedit provides semantic coding (especially UMLS codes) for data items and codelists. Is semantic coding available in iCRF?

 Minor comments

 page 2, Introduction, 2nd paragraph:

 HL7 is not a communication standard, but a standards developing organisation

 page 2, Introduction, 2nd paragraph: the "Meta Data Models website" correct term: Portal of Medical Data Models

 page 2, Introduction, 2nd paragraph: Link to Basic Health Data Set (available contents!) should be updated

 Figure 2 & Figure 4: should be shrinked, a lot of blank space

Are the conclusions about the tool and its performance adequately supported by the findings presented in the article?

Partly

Is the rationale for developing the new software tool clearly explained?

Yes

Is the description of the software tool technically sound?

Yes

Are sufficient details of the code, methods and analysis (if applicable) provided to allow replication of the software development and its use by others?

Yes

Is sufficient information provided to allow interpretation of the expected output datasets and any results generated using the tool?

Partly

Reviewer Expertise:

Medical Informatics, Medical Data Models, semantic annotations, interoperability

I confirm that I have read this submission and believe that I have an appropriate level of expertise to confirm that it is of an acceptable scientific standard, however I have significant reservations, as outlined above.

## References

[1] : Portal of medical data models: information infrastructure for medical research and healthcare. *Database (Oxford)*.2016;2016: 10.1093/database/bav121 10.1093/database/bav121 26868052PMC4750548

# Response

**Published:** 2020-03-16

**Authors:** Sander de Ridder

**Affiliations:** VU University Medical Center, Amsterdam, The Netherlands

Thank you reviewing of our paper. Please find below the changes we made to the new version to address your concerns/comments.

**Major comments**

**1. page 2, Methods, Supported Codebooks: Quantitative Information on 6 codebooks is missing: how many item definitions per codebook are available? how many codelist(items)?**

 We added this information to the “supported codebooks” section.

**2. page 2, Introduction: iCRF generates CRFs in Castor, OpenClinica3 and REDCap-Format.** **Why not CDISC ODM, which is endorsed by FDA? The Portal of Medical Data Models provides CRFs in 18 formats**

 One of the issues is that Castor doesn't support it yet. OpenClinica 3 doesn’t support direct import of ODM CRFs either. Hence, supporting ODM wasn’t a priority for us in the current release, but given your feedback, we added support for CDISC ODM to the roadmap. Accordingly, we updated: "If desirable, additional internationally established formats, such as CDISC ODM, may also be included in the future." in the “Additional EDCs” section to: "Support for CDISC ODM is on the roadmap. If desirable, additional internationally established formats may also be included in the future."

**3. page 3, Discussion "Manually mapping data definitions to a medical thesaurus such as SNOMED CT is complicated and time consuming"**

**=> Yes, this is absolutely correct. But it should be mentioned, that this mapping was already done for large sets of data items.**

**For example, the portal of medical data models provides ~520.000 data items with manual (physician-based) terminology mappings. The portal of medical data models is not just a website, it is a registered European information infrastructure.**

 We updated the manuscript to stress that many items have already been mapped.

**4. page 6, Additional EDCs: CDISC ODM is not just an internationally established format, it is required by regulatory authorities (FDA). Define XML (FDA) is based on CDISC ODM !**

 As mentioned in an earlier comment, CDISC ODM can be added, but it wasn't a priority for us. However, we added it to the roadmap.

**5. page 6, EDC-specific item customisation: processing of individual data items is a very work-intensive process, given the high number of data items in clinical studies (~ 500 - 2000). Re-Use of itemgroups (sets of data items) would be useful (this feature is available in ODMedit, which is covered in the discussion)**

 Thank you for the suggestion. We’d have to look into it more closely, but we added it as a possible new feature to the roadmap. 

**6. page 6, Discussion, similar work: It should be mentioned, that ODMedit provides semantic coding (especially UMLS codes) for data items and codelists. Is semantic coding available in iCRF?**

 The iCRF Generator is independent of the coding used. Hence, if a codebook there uses UMLS, the iCRF Generator will show UMLS codes. We added the following to the introduction:

 Hence, if a codebook is mapped to a medical thesaurus, the iCRF Generator allows the user to use these mappings, preventing the labour-intensive manual mapping.

**Minor comments**

**7. page 2, Introduction, 2nd paragraph - HL7 is not a communication standard, but a standards developing organisation**

 Health Level Seven International (HL7) is the standards developing organization. However, Health Level Seven (HL7) also refers to the standards. See e.g. their glossary here: https://www.hl7.org/documentcenter/public/calendarofevents/FirstTime/Glossary%20of%20terms.pdf

*Health Level Seven (HL7) is an application protocol for electronic data exchange in health care environments. The HL7 protocol is a collection of standard formats which specify the implementation of interfaces between computer applications from different vendors. This communication protocol allows healthcare institutions to exchange key sets of data amount different application systems. Flexibility is built into the protocol to allow compatibility for specialized data sets that have facility specific needs.*

 To avoid confusion, we updated the URL in the paper to directly link to the standards ( https://www.hl7.org/implement/standards/ ).

**8. page 2, Introduction, 2nd paragraph: the "Meta Data Models website" correct term: Portal of Medical Data Models**

 We apologise for the error. Fixed.

**9. page 2, Introduction, 2nd paragraph: Link to Basic Health Data Set (available contents!) should be updated**

 Thank for the suggestion. We added the URL.

**10. Figure 2 & Figure 4: should be shrinked, a lot of blank space**

 We removed blank space from the figures.

# Peer review: Reviewer response for version 1

**DOI:** 10.5256/f1000research.23777.r59542

**Published:** 2020-02-07

**Authors:** Rianne Fijten, Petros Kalendralis

**Affiliations:** 1 Department of Radiation Oncology (MAASTRO), GROW School for Oncology and Developmental Biology, Maastricht University Medical Centre (MUMC+), Maastricht, The Netherlands; 2 Department of Radiation Oncology (MAASTRO), GROW School for Oncology and Developmental Biology, Maastricht University Medical Centre (MUMC+), Maastricht, The Netherlands

**Overall**

 The authors' idea of creating an iCRF generator could alleviate pressure from data managers by making CRFs interoperable.

**Introduction**

 The authors have presented all relevant concepts needed to understand the rest of the article.

**Methods**

- **Codebooks**: Only 2 of 6 currently supported codebooks are generic enough to be used by users from a wide range of fields. Because the other four are all related to cancer, this could exclude a large group of users.
- **Creating the CRFs: **One of the arguments made in the article is that automating CRF will help people make CRFs more easily. However it is unclear whether using the CRF generator would actually save time. In fact, the figures imply that using the iCRF generator requires a lot of manual work as well.

**Figure 1**:

- In this figure, it is at first not clear where to start. We recommend using numbering to clarify the sequential steps.
- It is unclear from the figure and text how the workflow is structured. In fact, the figure seems to imply that there are three different workflows depending on the EDC that is selected by the user. This could be a potential hazard in terms of sustainability as adding an EDC would be a lot of work. A generic workflow with EDC-specific export functionalities would make the program much more future-proof.
- What does the summary show?

**Figure 3**

 To help users, we suggest to implement a way of providing information about the codebooks and their content. For example, a link to more information about each codebook or a dropdown view that shows the items in each codebook. This is not there currently and would require users to start searching for it themselves if they're not familiar (enough) with the content of the codebooks.

**Use cases**

 We would like to see a clearer example that also shows what each step in figure 1 looks like.

**Discussion**

- The discussion mentions that codebooks might be added when of sufficient quality. How do you define sufficient quality and who decides that?
- The authors mention that ODMedit's flexibility is a weakness compared to the iCRF generator. We believe that this could in fact be a strength if introduced in the iCRF generator. In fact, allowing users to contribute to the codebooks and item lists would widen the user base. To avoid the creation of faulty or multiple items denoting the same term, the authors could consider allowing users to contribute, but establish a curated group of codebooks and elements. This makes the software tool more flexible for its users.

Are the conclusions about the tool and its performance adequately supported by the findings presented in the article?

Partly

Is the rationale for developing the new software tool clearly explained?

Yes

Is the description of the software tool technically sound?

Partly

Are sufficient details of the code, methods and analysis (if applicable) provided to allow replication of the software development and its use by others?

Yes

Is sufficient information provided to allow interpretation of the expected output datasets and any results generated using the tool?

No

Reviewer Expertise:

Our area of research focuses on making medical data FAIR. For example, we employ the Personal Health Train to share information across medical centers in a privacy-preserving manner.

We confirm that we have read this submission and believe that we have an appropriate level of expertise to confirm that it is of an acceptable scientific standard, however we have significant reservations, as outlined above.

# Response

**Published:** 2020-03-16

**Authors:** Sander de Ridder

**Affiliations:** VU University Medical Center, Amsterdam, The Netherlands

Thank you reviewing of our paper. Please find below the changes we made to the new version to address your concerns/comments.

**Methods**

**1. Codebooks: Only 2 of 6 currently supported codebooks are generic enough to be used by users from a wide range of fields. Because the other four are all related to cancer, this could exclude a large group of users.**

 Indeed, currently the number of codebooks available is limited. We are hoping that by introducing the iCRF Generator to the public, this will stimulate them to publish codebooks in ART-DECOR, which we could then potentially make available in the iCRF Generator. Furthermore, Nictiz is also actively involved; they are currently looking into publishing e.g. established questionnaires.

 Given the comments here and later on, we rewrote the “Additional codebooks” paragraph in the discussion to:

 At this point, the iCRF Generator gives access to six nationally established codebooks, some of which support multiple languages and multiple versions. To improve the user-base for the iCRF Generator, the number and variety of codebooks available must increase. While nationwide standards, such as the Basic Health Dataset, can be readily made available, some form of governance may have to be put in place to establish other types of curated codebooks. This could stimulate the community to help build high-quality codebooks, mapped to medical thesauri, while preventing too much codebook redundancy and conflicting items. Furthermore, internationally established codebooks, such as CDISC’s CDASH can also be made available.

**2. Creating the CRFs: One of the arguments made in the article is that automating CRF will help people make CRFs more easily. However it is unclear whether using the CRF generator would actually save time. In fact, the figures imply that using the iCRF generator requires a lot of manual work as well.**

 It makes creating interoperable CRFs easier, not necessarily CRFs. Instead of having to e.g. define Gender and the appropriate codes and manually annotating with the appropriate thesaurus codes, which really takes a lot of time, instead you take the gender from the codebook and reuse that definition. However, you still need to define whether you would like have Gender as a radio button or a dropdown – that work does not change. We checked the manuscript and updated the Use cases section to stress this aspect:

 "When a decision has been made on what clinical data is going to be collected, the data manager has to design and build the CRFs for data collection. Instead of doing this manually, the iCRF Generator can be used to select items from the available codebooks and generate the basis of the case report forms."

 Rewritten to

 "When a decision has been made on what clinical data is going to be collected, the data manager has to design and build the CRFs for data collection. Instead of manually designing the items and mapping them to a medical thesaurus, which takes a lot of time, the iCRF Generator can be used to select items which have already been mapped from the available codebooks and generate the basis of the case report forms."

**3. Figure 1 - In this figure, it is at first not clear where to start. We recommend using numbering to clarify the sequential steps.**

 New version included in paper in which we numbered the steps

**4. It is unclear from the figure and text how the workflow is structured. In fact, the figure seems to imply that there are three different workflows depending on the EDC that is selected by the user. This could be a potential hazard in terms of sustainability as adding an EDC would be a lot of work. A generic workflow with EDC-specific export functionalities would make the program much more future-proof.**

 Indeed, it is a valid point that each EDC has its own customisation page, which thereby comes at the cost of maintainability / sustainability. However, this was done to facilitate the data managers. As an example, Castor's import file is an XML file, which you don't want to edit manually. If we remove all customisation options from de iCRF Generator, this implies all fields will have to be generated with default settings, or the XML will be invalid, e.g. every selection item will be a "required dropdown". This means that a datamanager would import these fields and would then have to manually edit all imported fields in Castor. We believe this would not make for a good user experience.

 We added the following to the "EDC-specific item customisation within iCRF Generator" section to clarify our reasoning:

 By adding this EDC-specific customisation, the iCRF Generator’s code is more difficult to maintain. However, by allowing the data manager to customise essential fields, the iCRF Generator can provide a ready-to-use CRF. This enhances the user experience, making it a worthwhile investment.

**5. What does the summary show?**

 In the text we mention the following about the summary: “The final page of the wizard shows a short summary of the number of selected items.” We’ve added a new Figure showing a screenshot of the summary.

**6. Figure 3 - To help users, we suggest to implement a way of providing information about the codebooks and their content. For example, a link to more information about each codebook or a dropdown view that shows the items in each codebook. This is not there currently and would require users to start searching for it themselves if they're not familiar (enough) with the content of the codebooks.**

 Great idea. We could change the page where you can select the codebooks and make the codebooks themselves e.g. clickable, linking to their ART-DECOR page. We'll add this to the roadmap for a future release.

**Use cases**

**7. We would like to see a clearer example that also shows what each step in figure 1 looks like.**

 To clarify the relationship between Figure 1 and the example figures (2-6) we updated the text to show how each step in Figure 1 is related to the example figures:

 Figure 1 illustrates the iCRF Generator’s complete workflow and figure 2-6 show actual examples of the workflow. In a typical use case, the user first selects an EDC from the dropdown ( Figure 1, step 1; Figure 2). The user then clicks the “Run” button, after which the wizard interface is started. The first wizard page asks the user to select one or more codebooks (Figure 1, step 2; Figure 3). <etc>

**Discussion**

**8. The discussion mentions that codebooks might be added when of sufficient quality. How do you define sufficient quality and who decides that?**

 Our initial aim was to provide a tool which can facilitate the reuse of the available codebooks. How to provide the users with high-quality codebooks is open for discussion and we welcome any input on the matter. We did rewrite this section though - see point 1.

**9. The authors mention that ODMedit's flexibility is a weakness compared to the iCRF generator. We believe that this could in fact be a strength if introduced in the iCRF generator. In fact, allowing users to contribute to the codebooks and item lists would widen the user base. To avoid the creation of faulty or multiple items denoting the same term, the authors could consider allowing users to contribute, but establish a curated group of codebooks and elements. This makes the software tool more flexible for its users.**

 Any codebook can be added to ART-DECOR, as Nictiz welcomes any additions. To facilitate interoperability, we ourselves foremost welcome any (inter)nationally endorsed standard, such as the Basic Health Dataset. We agree that establishing a curated group of codebooks which are properly mapped to medical thesauri would be great, although this does require a proper decision-making structure (governance) as well as the appropriate funding. We rewrote this section - see point 1.
